# Surgical Management of Thumb Ulnar Collateral Ligament Injuries: A Systematic Review and Meta-analysis of 614 Patients With a Minimum 1 Year Follow-up

**DOI:** 10.5435/JAAOSGlobal-D-25-00082

**Published:** 2025-05-02

**Authors:** Adam Schumaier, Francine Zeng, Chris McCarthy

**Affiliations:** From the OrthoCincy, KY/OH (Dr. Schumaier); The Hand Center, CT (Dr. Schumaier, Dr. McCarthy); the Hartford Healthcare, CT (Dr. Schumaier, Dr. Zeng, Dr. McCarthy); and the University of Connecticut, CT (Dr. Schumaier, Dr. Zeng, Dr. McCarthy).

## Abstract

**Background:**

Thumb ulnar collateral ligament (UCL) injuries are relatively common and may lead to pain and instability. Outcomes following surgical management have generally been acceptable, but data comparing techniques are limited. Furthermore, the optimal timeframe for surgery has not been established. The purpose of this systematic review and meta-analysis was to compare outcomes of different surgical techniques and timeframes for treating thumb UCL injuries.

**Methods:**

This study was conducted according to the Preferred Reporting Items for Systematic Reviews and Meta-Analyses guidelines. Data were analyzed based on the type of surgery performed: primary repair (PR) to soft tissue or bone tunnels, suture anchor repair (SAR) with or without internal brace, or reconstruction (REC) with autograft. A random effects model was used.

**Results:**

The analysis included 24 studies with 616 thumbs (PR = 146, SAR = 371, REC = 99). Average follow-up was 47 months. Average time from injury to surgery was 9.3 days (PR), 4.1 months (SAR), and 19.1 months (REC). The most commonly stated indication for surgery was lack of a firm end point on collateral stress testing. In the reconstruction group, all injuries were described as chronic or irreparable. A notable difference was found in the proportion of stable thumbs (PR 95%, SAR 95%, REC 81%) and return to unrestricted activities (PR 96%, SAR 93%, REC 84%). No clinically notable differences were observed in pain, grip strength, pinch strength, QuickDASH scores, return to work, complications, or complications requiring intervention.

**Conclusion:**

Surgical management of thumb UCL injuries produces overall favorable results. Acute, subacute, and repairable injuries treated with primary repair or suture anchor repair are more likely to be stable and allow unrestricted return to prior activities compared with chronic, irreparable injuries treated with reconstruction.

Injuries of the thumb ulnar collateral ligament (UCL) are relatively common and may lead to pain and instability. Thumb UCL injuries generate approximately 50 out of every 100,000 visits to emergency centers^1^ and most frequently occur in athletes or working age adults. It was first described as a chronic, repetitive injury in rabbit hunters and was termed “gamekeeper's thumb.” Later, it was described as an acute sports injury and was termed “skier's thumb.”^2^ Both descriptions suggested forced abduction and hyperextension as the mechanism of injury, with patients often experiencing pain and difficulty with pinch and grasp.

Treatment of thumb ulnar collateral ligament injuries is variable. Most authors suggest nonsurgical treatment with bracing, splinting, or casting for partial tears with surgery reserved for complete tears and those who fail nonsurgical management. Several surgical treatments have been described. Acute and subacute “repairable” injuries are often treated with primary repair of the ligament stumps,^3,4^ repair or advancement to adjacent soft tissue,^3,5,6^ and repair through bone tunnels^4,6^ or suture anchors.^3,7-18^ Some authors recommend augmentation of the repair using suture tape as an internal brace.^7,9^ Chronic “irreparable” injuries are often treated with reconstruction using a range of autografts, such as such as palmaris longus,^19-23^ flexor carpi radialis,^20,21,23^ plantaris,^21^ toe extensors,^21^ or extensor retinaculum.^24^ Some authors describe bone-tendon, bone-retinaculum, or bone-periosteum hybrid grafts from the wrist^13,25^ or iliac crest.^26^

Outcomes following surgical management have generally been acceptable, but data comparing techniques are limited. Furthermore, the optimal timing of surgical treatment for thumb UCL injuries has not been defined. The purpose of this systematic review and meta-analysis was to compare outcomes of different surgical techniques and timeframes for treating thumb UCL injuries. The hypothesis was that primary repair, suture anchor repair, and reconstruction would have comparable rates of stability and return to activities with no notable difference in patient-centered outcomes.

## Methods

**Figure 1:**
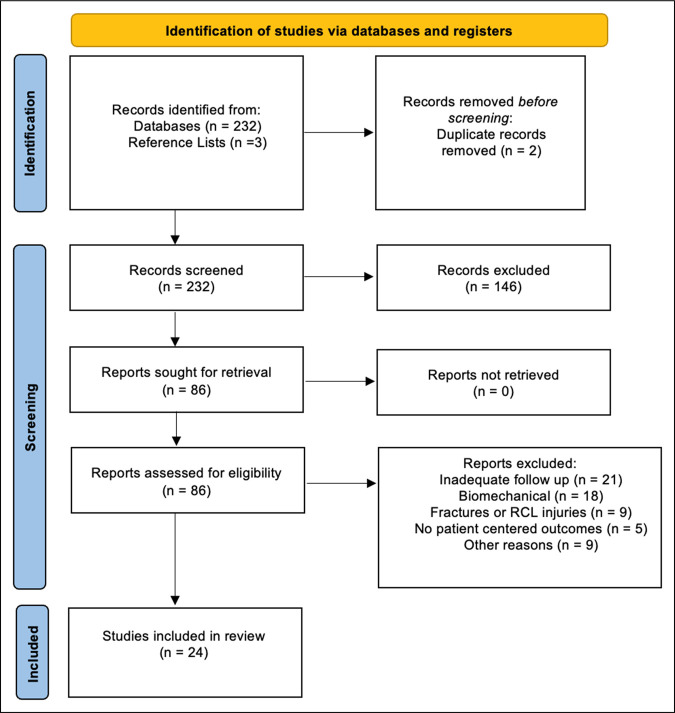
PRISMA flowchart demonstrating study selection. PRISMA = Preferred Reporting Items for Systematic Reviews and Meta-Analyses.

This study is a systematic review and meta-analysis performed according to the Preferred Reporting Items for Systematic Reviews and Meta-Analyses guidelines. A literature search of the PubMed, National Center for Biotechnology Information (NCBI), and Cochrane databases was done in September 2023. The following search term was used: ([thumb] AND ([ulnar collateral ligament] OR [UCL])) OR (Gamekeeper's thumb) OR (Skier's thumb) AND ([reconstruction] OR [repair]). A date range was not specified. Human clinical studies available in English and reporting the results of repair or reconstruction of the thumb ulnar collateral ligament in skeletally mature individuals were considered for inclusion. After removal of duplicates, 232 abstracts were identified and reviewed. Of these 232 abstracts, 86 articles were selected for full-text review with an additional three articles identified from reference lists. A total of 24 studies met final inclusion (Figure 1).^3-23,25-27^ The final inclusion criteria required a description of the surgical technique, an assessment of the time from injury to surgery, a minimum 1-year follow-up, an assessment of thumb stability, and at least one quantifiable patient-centered or patient-reported outcome. Avulsion fractures that were treated with fracture fixation were excluded. The studies were assessed by two authors and scored for quality using the Methodological Index for Non-Randomized Studies (MINORS).^28^

The following demographic data were collected: number of patients treated, number of thumbs treated, sex, age, time from injury to surgery, and duration of follow-up. The following perioperative data were collected: indications for surgery, surgical technique, postoperative immobilization, and postoperative activity restrictions. The primary outcome was thumb stability, which was categorized into thumbs that were stable, mildly lax, or unstable. A stable thumb was stable by the patient's assessment, stable by the examiner's assessment, or had less than 10 degrees of laxity. A mildly lax thumb had mild or increased laxity with a firm end point or had 10 to 20 degrees of laxity. An unstable thumb was unstable by the patient's assessment, unstable by the examiner's assessment, or had greater than 20 degrees of laxity. This categorization was selected to accommodate both qualitative and quantitative assessments and is based on the Glickel scoring system.^19-22,25^ Stability is routinely assessed in comparison to the contralateral side; when an absolute degree of laxity was reported, all but one study explicitly stated that the measurement was in comparison to the contralateral. The following secondary outcomes were collected: pain on a visual analog scale (VAS), pain on a qualitative scale, grip strength as a percentage of contralateral, pinch strength as a percentage of contralateral, return to work, return to sports and unrestricted activities, QuickDASH scores, complications, and complications requiring intervention.

Data were analyzed based on the type of surgery performed: primary repair (PR) to soft tissue or bone tunnels, suture anchor repair (SAR) with or without internal brace, or reconstruction (REC) with autograft. Means, proportions, and standard deviations were obtained for pooling of effect sizes for meta-analysis. When these parameters were not reported in the article, corresponding authors were contacted for the information. If no response was obtained following two separate contact attempts or the authors did not have the data, then the means and standard deviations were estimated using the medians, ranges, and confidence intervals according to Hozo^29^ and the Cochrane group.^30^ If none of these methods were successful, then the data were excluded from analysis.

Given the heterogeneity among studies, a conservative analysis was used to produce wide confidence intervals and minimize the likelihood of false-positives.^31,32^ Data were analyzed using a random effects model with inverse variance pooling and a generalized, linear, mixed-effects model with log transformation of proportions. A restricted maximum-likelihood estimator for τ^2^ was used (assuming a common τ^2^ among subgroups).^33^ Hartung-Knapp adjustments were used to calculate the confidence intervals.^31,34^ Heterogeneity was quantified with the *I*^2^ statistic.^35^ A *P* value of <0.05 was considered notable. Data were analyzed using R version 4.3.2 (R Foundation for Statistical Computing, Vienna, Austria) with RStudio version 2023.9.1.494 (RStudio Inc., Boston, Massachusetts). The following R packages were used: meta version 6.5-0 (Balduzzi, Rücker, and Schwarzer)^36^ and metafor version 4.4-0 (Viechtbauer).^37^

## Results

A total of 614 patients and 616 thumbs were included from the 24 studies. The average duration of follow-up was 47.2 months (range 11 to 247 months, range of study averages 19-79 months). The studies were either level II (2), level III (9), or level IV (13) evidence. The average MINORS score was 83% (±8%, range 66% to 97%), indicating relatively strong study quality (Supplementary Table, http://links.lww.com/JG9/A407). A total of two radial collateral ligament injuries (1 each in the PR and REC groups) were unable to be isolated from an otherwise high-quality comparative study with a MINORS score of 96%.^13^ The average patient age (40 years) and ratio of male to female patients (58:42) was similar among the groups. A quantifiable time from injury to surgery was reported in 19 out of 24 studies and averaged 9.3 days in the PR group, 4.1 months in the SAR group, and 19 months in the REC group (Table 1). The most commonly stated indication for surgery was the lack of a firm end point on collateral stress testing (14 out of 24 studies). In the reconstruction group, all injuries were described as chronic or irreparable, and the shortest average time from injury to surgery was 5.2 months. A total of 31 patients from two studies in the SAR group were treated with suture tape augmentation.^7,9^ The type and duration of postoperative immobilization, postoperative restrictions, and postoperative therapy were unique for each study. Most authors used a spica splint or cast in the immediate postoperative period followed by transition to a removable brace that was discontinued between 4 and 12 weeks postoperatively.

**Table 1:** Demographics (n = 614 Patients)

	Primary Repair	Suture Anchor Repair	Reconstruction
n Thumbs	146	371	99
Sex (% male)	54%	58%	61%
Age	40 y (30-45)	41 y (20-50)	38 y (29-53)
Injury to surgery	9.3 d (2.7 d-11.3 d)	4.1 m (1.5 d-32 m)	19.1 m (5.2 m-42.3 m)
Follow-up	39.7 m (33.7-45.8)	49.0 m (19.1-78.9)	49.3 m (28.0-70.5)

Data presented as means (range of means), d = days, m = months, y = years.

**Figure 2:**
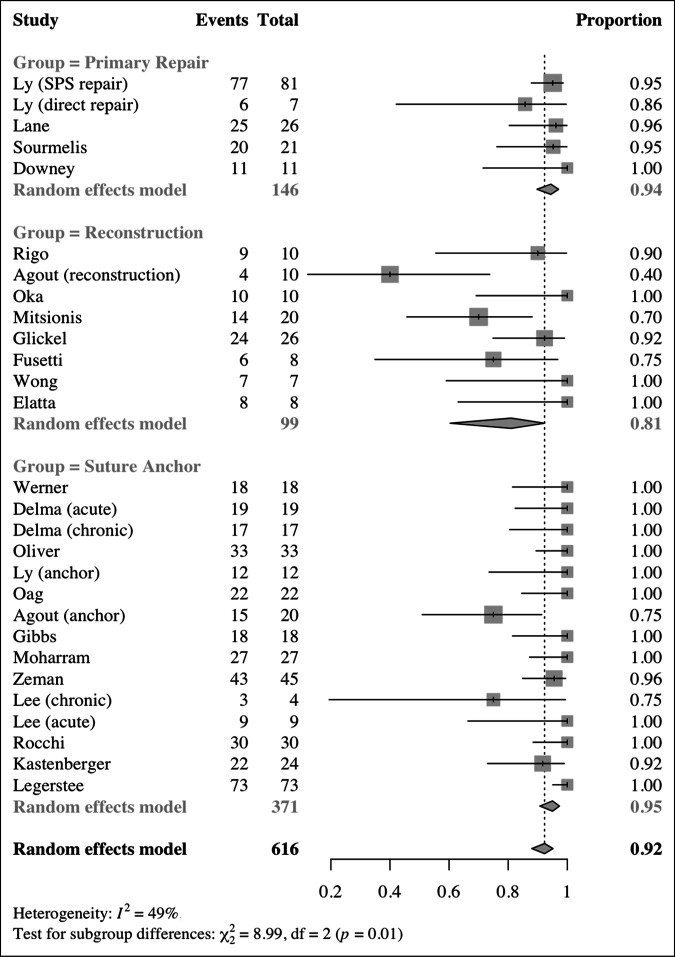
Forest plot showing the proportions of postoperative stable thumbs

The proportion of postoperative stable thumbs was higher in the suture anchor (95%) and primary repair groups (94.5%) compared with the reconstruction group (81.1%) (*P* = 0.011, *I*^2^ = 49.0%). The proportion of mildly lax and unstable thumbs was higher in the reconstruction group (16.2%, 8.5%) compared with the suture anchor (3.9%, 3.3%) and primary repair groups (5.2%, 2.8%) (*P* = 0.016, *I*^2^ = 42.2%, *P* = 0.003, *I*^2^ = 0%) (Table 2, Figure 2). Statistically significant differences were seen in VAS pain scores, grip strength, and tip pinch, but these differences are unlikely to be clinically important (Table 3). No notable difference was found in pain on a qualitative scale, key pinch, or QuickDASH scores (Table 3). Return to work was 96% or higher and similar among groups (*P* = 0.95, *I*^2^ = 0%); however, the rate of return to sports and unrestricted activities was higher in the repair (96%) and suture anchor repair groups (93%) compared with the reconstruction group (84%) (*P* = 0.012, *I*^2^ = 0%). No difference was observed in the rate of complications (12%, *P* = 0.125, *I*^2^ = 46.2%) or complications requiring intervention (1%, *P* = 0.961, *I*^2^ = 0%) (Table 4). The most common complications were radial sensory or digital neurapraxia (n = 24) and complex regional pain syndrome (n = 10).

**Table 2:** Estimates of Thumb Stability (n = 616 Thumbs)

	Primary Repair	Suture Anchor Repair	Reconstruction	*P*	I^2^
Stable	94.5% (89.7-97.1%)	95% (90.7%-97.4%)	81.1% (60.4-92.3%)	0.011	49.0%
Mild laxity	5.2% (2.3-11.4%)	3.9% (2.0-7.5%)	16.2% (6.3-36.0%)	0.016	42.2%
Unstable	2.8% (1.0-7.8%)	3.3% (2.2-5.0%)	8.5% (4.9-14.4%)	0.003	0.0%

Mean (95% confidence interval).

**Table 3:** Estimates of Pain and Strength

	Primary Repair	Suture Anchor	Reconstruction	*P*	I^2^
Pain (VAS, n = 414)	2.2 (1.1-4.4)	1.1 (0.6-1.9)	1.3 (0-10)	0.036	80.9%
Pain (qualitative, n = 178)					
None	77% (39.9-94.4%)	82.5% (0-100%)	81.2% (68.2-89.7%)	0.953	52.7%
Mild intermittent	23.1% (5.8-59.3%)	14.5% (0-100%)	18.9% (10.4-31.9%)	0.907	26.8%
Often moderate	1.9% (0.1-2.4%)	4.3% (0-96.1%)	3.5% (1.9-6.2%)	0.818	0.0%
Grip (n = 151)	81.8% (52.8-110.7%)	95.3% (82.4-108.2%)	91.1 (72.8-109.5%)	< 0.001	69.4%
Tip pinch (n = 168)	80.8% (70.6-91.0%)	87.0% (82.2-91.9%)	89.2% (85.6-92.8%)	< 0.001	10.1%
Key pinch (n = 136)	78.9% (11.2-147%)	81.6% (62.4-100.8%)	90.9% (8.0-173.9%)	0.353	73.3%

Mean (95% confidence interval).

**Table 4:** Estimates for Patient-Reported Outcomes and Complications

	Primary Repair	Suture Anchor	Reconstruction	*P*	I^2^
Return to work (n = 212)	95.6% (84.3-98.9%)	97.3% (90.0-99.3%)	97.0% (74.4-99.7%)	0.828	0.0%
Return to sports/activities (n = 354)	97.4% (91.2-99.3%)	95.4% (90.4-97.9%)	84.5% (72.6-92.2%)	0.021	0.0%
QuickDASH (n = 249)	8.8 (5.7-11.9)	5.1 (1.0-9.2)	8.1 (0-100)	0.094	85.6%
Complications (n = 575)	10.3% (3.7-25.8%)	10.4% (6.0-17.5%)	25.7% (11-47.9%)	0.125	46.2%
Requiring intervention	1.2% (0.1-9.9%)	1.2% (0.2-5.6%)	0.8% (0.1-10.9%)	0.961	0.0%

Mean (95% confidence interval).

## Discussion

Thumb UCL injuries are relatively common and may lead to activity limiting pain or instability. The purpose of this systematic review and meta-analysis was to compare the outcomes of different surgical techniques and timeframes for treating thumb UCL injuries. The results of surgical management were overall favorable, with most patients achieving a stable thumb with high rates of return to work and prior activities. However, the results suggest that acute, subacute, and repairable injuries treated with repair or suture anchor repair are slightly more likely to produce a stable thumb that permits return to sports and unrestricted activities compared with chronic, irreparable injuries treated with ligament reconstruction. Injuries treated with repair also avoid the extra surgical morbidity associated with autograft harvest.

An earlier systematic review by Samora et al.^38^ in 2013 evaluated 14 studies that compared nonsurgical treatment, repair, and reconstruction of thumb UCL injuries. The authors did not find any differences between repair and reconstruction for acute or chronic injuries. Although a meta-analysis was not done, their results suggested that surgical management is effective regardless of the method or timing of intervention. This is in contrast to this study, which suggests that the results of early repair are slightly more favorable than reconstruction of chronic injuries. Notably, 13 of the studies included in this analysis were published after the work by Samora et al. in 2013, which likely explains some of the differences.

A 2024 meta-analysis by Legerstee et al.^14^ evaluated 29 studies and compared the outcomes of primary suture repair, suture anchor fixation, Kirschner wire fixation, and a combination of soft-tissue techniques. The outcomes of ligament reconstruction were not evaluated; however, no clinically notable differences were observed between the different repair techniques, and most patients had stable thumbs (99%) without pain (87%). Avulsion fractures were included in their analysis, and a minimum follow-up duration was not specified. Despite these differences in methodology, their results are comparable to this study and further suggest that outcomes are similar among ligament repair techniques.

The difference in outcomes between repairs and reconstructions is likely due to the chronicity of the injury. Injuries in the reconstruction group were all described as chronic or irreparable, and surgeons typically use reconstruction only in cases with poor tissue quality when there is no other choice. The time from injury to surgery in the reconstruction group averaged 19.1 months, which is considerably longer than the 9.3 days and 4.1 months in the primary repair and suture anchor repair groups. It is also possible that chronic UCL insufficiency leads to attenuation of other stabilizing structures like the joint capsule or volar plate, which would not be addressed by ligament reconstruction. Disuse may lead to weakening of the dynamic stabilizers. Another possibility is that ligament reconstruction requires healing at two separate grafting sites to restore stability. In comparison, a repairable ligament only requires healing at the site of disruption, and the healing environment might be more favorable in an acute or subacute setting. The timeframe for repairability is not clear, but the average time from injury to surgery for suture anchor repair ranged from 1.5 days to 32 months. A prospective study included in this analysis evaluated 76 thumbs at 1 year and did not find any notable difference in outcomes among those who underwent repair within 3 weeks, between 3 and 6 weeks, or between 6 weeks and 6 months.^14^ This suggests that delayed repair or an initial trial of nonsurgical management is unlikely to negatively affect long-term functional outcomes.

Adequate results of nonsurgical treatment have been reported for both avulsion fractures and complete ligamentous ruptures. An earlier study^39^ included 30 patients with UCL avulsion fractures with displacement up to 1.5 mm and less than 30% articular involvement who were treated with thumb-spica immobilization. At a mean 3-year follow-up, 34% remained symptomatic, 16% remained unstable, and 25% had nonunions. Despite this, all patients were satisfied with their outcome. Another earlier study^40^ evaluated nonsurgical management with 8 to 12 weeks of thumb spica splint immobilization in 40 thumbs with complete ligamentous UCL ruptures at a minimum 1-year follow-up. In total, 85% of the patients healed without instability, and only 15% demonstrated persistent instability at 12 weeks, which was subsequently treated surgically.

There are several limitations to this study. The patients represent a heterogenous population with a wide age range and variable functional demands. The postsurgical protocols, including physical therapy and duration of immobilization, were unique to each study. Only four of the 24 studies included a comparison group. Although the patients were grouped into primary repair, suture anchor repair, and reconstruction, the surgical technique within each group and within each study was variable. For example, the number of tunnels used, utilization of Kirschner wires or suture buttons, type of suture material, type of anchor, and the type of autograft was too variable to permit a satisfactory subanalysis. In addition, the assessment of thumb stability was not uniform among the studies and included a mixture of quantitative and qualitative assessments, and the interrater reliability of assessing thumb stability has not been established. Finally and most importantly, the surgical technique was inseparable from the time from injury to surgery. Unfortunately, there was insufficient patient-level data to permit a subanalysis based on the time to surgery. The results of this study do not suggest that all injuries should be repaired.

In conclusion, this systematic review and meta-analysis of 614 patients with a minimum 1-year follow-up suggests that surgical management of thumb UCL injuries produces overall favorable results. Acute, subacute, and repairable injuries treated with primary repair or suture anchor repair are more likely to be stable and allow unrestricted return to sports and prior activities compared with chronic, irreparable injuries treated with reconstruction.

## Supplementary Material
