# Supplementary material for: Surgical Management of Thumb Ulnar Collateral Ligament Injuries: A Systematic Review and Meta-analysis of 614 Patients With a Minimum 1 Year Follow-up
Source: J Am Acad Orthop Surg Glob Res Rev. 2025 May 2;9(5):e25.00082. doi: 10.5435/JAAOSGlobal-D-25-00082 (PMC12052237; doi:10.5435/JAAOSGlobal-D-25-00082)
Supplement: Supplementary file 1 [file jagrr-9-e25.00082-s001.docx]

| Author | PMID | Year | Design | LOE | Scores | | | | | | |  |  |  |  |  |  |  |  |  |  |  |  |  |  |
| --- | --- | --- | --- | --- | --- | --- | --- | --- | --- | --- | --- | --- | --- | --- | --- | --- | --- | --- | --- | --- | --- | --- | --- | --- | --- |
|  |  |  |  |  | Rater 1 | | Rater 2 | | Average | | |  |  |  |  |  |  |  |  |  |  |  |  |  |  |
| Agout | 28576699 | 2017 | retrospective comparative series | 3 | 83.3% | | 79.2% | | 81.3% | | |  |  |  |  |  |  |  |  |  |  |  |  |  |  |
| Delma | 35601522 | 2022 | retrospective comparative series | 3 | 91.7% | | 83.3% | | 87.5% | | |  |  |  |  |  |  |  |  |  |  |  |  |  |  |
| Downey | 7778709 | 1995 | retrospective case series | 4 | 87.5% | | 87.5% | | 87.5% | | |  |  |  |  |  |  |  |  |  |  |  |  |  |  |
| Elatta | 37357784 | 2023 | case series | 4 | 87.5% | | 87.5% | | 87.5% | | |  |  |  |  |  |  |  |  |  |  |  |  |  |  |
| Fusetti | 16277145 | 2005 | case series | 4 | 87.5% | | 81.3% | | 84.4% | | |  |  |  |  |  |  |  |  |  |  |  |  |  |  |
| Gibbs | 32775473 | 2020 | retrospective case series | 4 | 56.3% | | 75.0% | | 65.6% | | |  |  |  |  |  |  |  |  |  |  |  |  |  |  |
| Glickel | 8228072 | 1993 | retrospective case series | 4 | 87.5% | | 81.3% | | 84.4% | | |  |  |  |  |  |  |  |  |  |  |  |  |  |  |
| Kastenberger | 33040206 | 2021 | prospective case series | 3 | 93.8% | | 93.8% | | 93.8% | | |  |  |  |  |  |  |  |  |  |  |  |  |  |  |
| Katolik | 18971729 | 2008 | retrospective comparative series | 3 | 81.3% | | 75.0% | | 78.1% | | |  |  |  |  |  |  |  |  |  |  |  |  |  |  |
| Lane | 1867332 | 1991 | case series | 4 | 75.0% | | 75.0% | | 75.0% | | |  |  |  |  |  |  |  |  |  |  |  |  |  |  |
| Lee | 32000599 | 2020 | retrospective comparative series | 3 | 75.0% | | 75.0% | | 75.0% | | |  |  |  |  |  |  |  |  |  |  |  |  |  |  |
| Legerstee | 37294237 | 2023 | prospective comparative series | 2 | 95.8% | | 95.8% | | 95.8% | | |  |  |  |  |  |  |  |  |  |  |  |  |  |  |
| Ly | 36764359 | 2023 | retrospective comparative series | 3 | 83.3% | | 79.2% | | 81.3% | | |  |  |  |  |  |  |  |  |  |  |  |  |  |  |
| Mitsionis | 11062584 | 2000 | case series | 4 | 81.3% | | 81.3% | | 81.3% | | |  |  |  |  |  |  |  |  |  |  |  |  |  |  |
| Moharram | 24126337 | 2013 | prospective case series | 3 | 87.5% | | 87.5% | | 87.5% | | |  |  |  |  |  |  |  |  |  |  |  |  |  |  |
| Oag | 34864217 | 2022 | prospective case series | 3 | 87.5% | | 81.3% | | 84.4% | | |  |  |  |  |  |  |  |  |  |  |  |  |  |  |
| Oka | 12923939 | 2003 | case series | 4 | 75.0% | | 75.0% | | 75.0% | | |  |  |  |  |  |  |  |  |  |  |  |  |  |  |
| Oliver | 36476090 | 2022 | retrospective case series | 4 | 87.5% | | 81.3% | | 84.4% | | |  |  |  |  |  |  |  |  |  |  |  |  |  |  |
| Rigo | 24922328 | 2014 | case series | 4 | 81.3% | | 68.8% | | 75.0% | | |  |  |  |  |  |  |  |  |  |  |  |  |  |  |
| Rocchi | 24185690 | 2014 | randomized trial | 2 | 93.8% | | 100.0% | | 96.9% | | |  |  |  |  |  |  |  |  |  |  |  |  |  |  |
| Sourmelis | 9385267 | 1997 | case series | 4 | 75.0% | | 68.8% | | 71.9% | | |  |  |  |  |  |  |  |  |  |  |  |  |  |  |
| Werner | 25139463 | 2014 | retrospective case series | 4 | 81.3% | | 81.3% | | 81.3% | | |  |  |  |  |  |  |  |  |  |  |  |  |  |  |
| Wong | 19181232 | 2009 | prospective case series | 3 | 87.5% | | 100.0% | | 93.8% | | |  |  |  |  |  |  |  |  |  |  |  |  |  |  |
| Zeman | 9784810 | 1998 | case series | 4 | 68.8% | | 75.0% | | 71.9% | | |  |  |  |  |  |  |  |  |  |  |  |  |  |  |
| PMID = pubmed identification number, LOE = level of evidence | | | |  |  |  | |  | |  |  | |  |  |  |  |  |  |  |  |  |  |  |  |  |
